# A Severe Case of Diabetic Lumbosacral Radiculoplexus Neuropathy: A Diagnosis of Exclusion

**DOI:** 10.7759/cureus.61969

**Published:** 2024-06-08

**Authors:** Kevin T Dao, Johar Leepakshi, Jesslin Abraham, Cesar M Aranguri, Matthew Clarke, Britney T Ly, Hari K Veedu, Katayoun Sabetian

**Affiliations:** 1 Internal Medicine, University of California Los Angeles-Kern Medical Center, Bakersfield, USA; 2 Internal Medicine, Western University of Health Sciences, Pomona, USA; 3 Neurology, University of California Los Angeles-Kern Medical Center, Bakersfield, USA

**Keywords:** exclusion, differential diagnoses, neuropathic, non-systemic microvasculitis, diabetic amyotrophy, diabetic lumbosacral radiculoplexus neuropathy

## Abstract

Diabetic lumbosacral radiculoplexus neuropathy (DLSRPN), also known as diabetic amyotrophy, is a rare disease of exclusion that is difficult to diagnose due to its non-specific clinical presentation of neuropathy, autonomic symptoms, and potential weight loss. Due to this, many differential diagnoses are raised before making a diagnosis of such an uncommon disease. However, once the diagnosis is made, the management of this disease can vary. Here, we would like to discuss the etiology, pathophysiology, diagnosis, and management of this disease, as well as present a rare case of diabetic lumbosacral radiculoplexus neuropathy in a 50-year-old male.

## Introduction

Diabetic lumbosacral radiculoplexus neuropathy (DLSRPN) is a type of proximal diabetic neuropathy caused by a variety of mechanisms that usually presents with unilateral pelvic-femoral pain, weakness, and atrophy [1]. Patients with this disease initially present with symptoms that occur in the proximal portions of the lower extremities, particularly the thigh, which then spread to the contralateral limb distally or proximally after a period of many weeks or even months [1,2]. Despite it being called diabetic lumbosacral radiculoplexus neuropathy, patients can have weakness in the thoracic areas and upper limbs, leading to weakness in the upper extremities [3,4]. Due to the unorthodox progression of this disease, several hypotheses have been suggested regarding the etiology of DLSRPN, including ischemic, metabolic, inflammatory, etc. [5]. However, studies have assumed that the most likely cause of DLSRPN is an ischemic nerve injury from a non-systemic microvasculitis associated with other symptoms, particularly weight loss, autonomic symptoms, and type 2 diabetes [5]. Despite these non-specific symptoms, it is considered a rare disorder, affecting only 1% of diabetic patients [6] around 65-70 years old. Unfortunately, due to the common clinical features and low incidence of this disease, diagnosing DLSRPN can be quite challenging. Therefore, we would like to present a patient with DLSRPN as well as the various diagnoses to consider, abnormal laboratory values, and when to make such a diagnosis.

The article was previously presented as an iPoster at Southern San Joaquin Valley Research Forum on May 25, 2023. The article was previously presented as an iPoster at Solomon Scholars Program on June 5, 2023. The article was previously presented as a presentation at the Western Medical Research Conference (AFMR) on January 18, 2024.

## Case presentation

A 50-year-old Caucasian wheelchair-bound male with a past medical history of type 2 diabetes and perineal abscess presents to the emergency department, following a neurology clinic visit due to complaints of bilateral lower and upper extremity weakness with gradual, constant, and non-radiating pain without any alleviating or aggravating factors. He says that his initial symptoms began with his left proximal thigh, slowly progressing to his right proximal thigh, with eventual progression to his proximal upper extremities. He stated that he had lost 60 to 65 pounds in the last few months and had become increasingly fatigued, spending the majority of his time bedbound. He denies any genitourinary symptoms and states that his bilateral lower extremity weakness has gotten worse within the past few weeks. It is worth noting that he has a long history of smoking 12 cigarettes per day for many years, but he is unable to specify how many. A stat lumbar puncture was requested by the neurology clinic as well as treatment with intravenous immunoglobulin (IVIG) 400 mg/kg.

**Figure 1:**
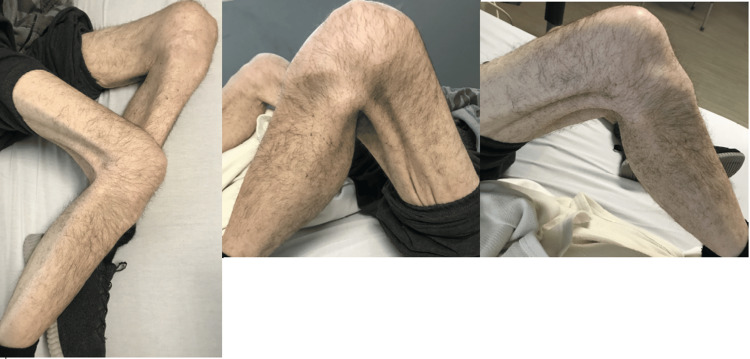
Patient's bilateral lower extremities on admission. The figure shown depicts the patient's bilateral lower extremities that were seen on physical exam during admission. Of note, there were areas of muscle wasting and atrophy noted.

At the initial presentation, he was wheelchair-bound and unable to stand with support. Vitals showed a temperature of 37.1°C, a heart rate of 86 beats per minute, a respiratory rate of 22 breaths per minute, a blood pressure of 130/77 mmHg, and a SpO_2_ of 98%. A physical exam revealed mildly atrophic bilateral lower extremities, with further details in Figure 1. Further examination reveals that the bilateral upper extremities had +4/5 motor functions with an American Spinal Injury Association (ASIA) sensory score of 2, and the bilateral lower extremities had an ASIA sensory score of 1 with +3/5 motor functions in both the proximal and distal bilateral lower extremities. However, the range of motion was limited due to pain and weakness, with further details noted in Table 1. Deep tendon reflexes were absent or decreased in the bilateral upper and lower extremities. The rest of the physical exam, including the heart, head, neck, ears, throat, lungs, and abdomen, was unremarkable.

**Table 1:** Physical exam of extremity motor function on admission. The table shows a physical exam assessment of the patient's motor functions based on the motor function scale with +0/5 equating to no muscle contraction, +1/5 equating to trace of contraction, +2/5 equating to contraction but not against gravity, +3/5 equating to movement against gravity but not resistance, +4/5 equating to movement with some weakness against resistance, and +5/5 equating to normal motor function.

Extremity	Strength
Left upper extremity	+4/5
Right upper extremity	+4/5
Left hip flexion	+2/5
Left knee flexion	+2/5
Left knee extension	+2/5
Left ankle dorsi flexion	+0/5
Left ankle plantar flexion	+3/5
Left great toe extension	+0/5
Right hip flexion	+2/5
Right knee flexion	+2/5
Right knee extension	+2/5
Right ankle dorsi flexion	+0/5
Right ankle plantar flexion	+3/5
Right great toe extension	+0/5

**Figure 2:**
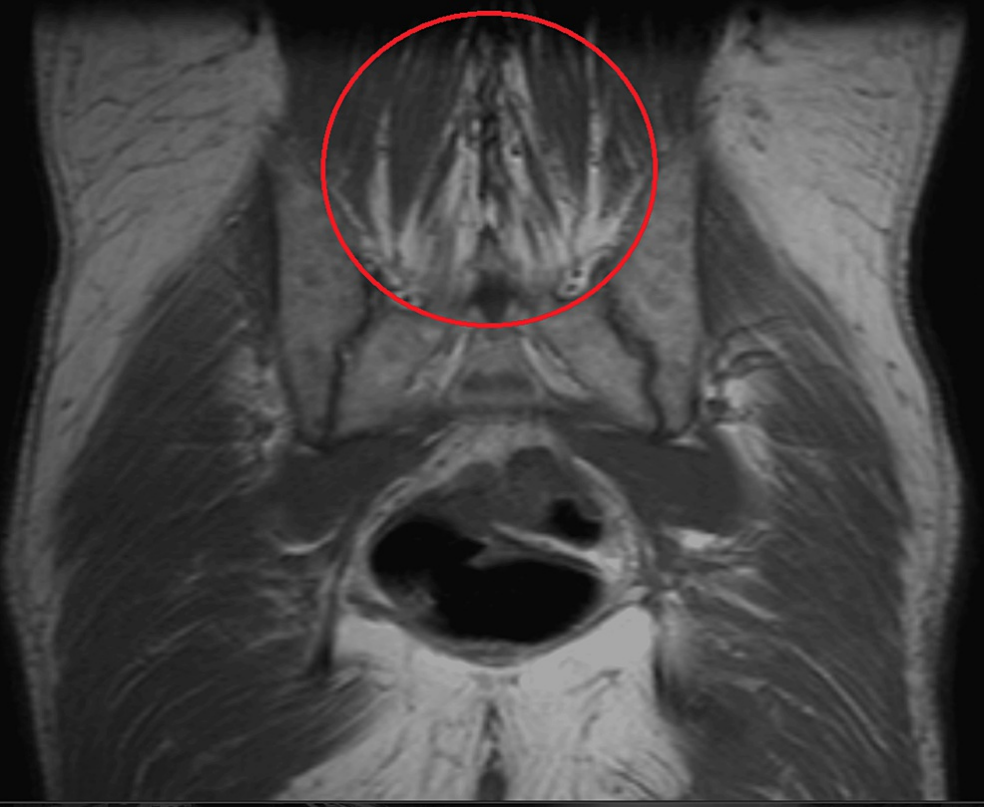
MRI of pelvis without contrast. The red circle demonstrates an increased signal in the paraspinal muscles and bilateral abductor muscles without any evidence of masses or fluid collections.

The patient’s basic metabolic panel showed a mildly elevated HbA1c of 6.5% with an unremarkable glucose level of 151 mg/dL. All other blood work, such as complete blood count, with mean corpuscular volume in the 80s, autoimmune titers (Table 2), and urine toxicology screen, were unremarkable. The chest X-ray showed no evidence of active cardiopulmonary disease. Lumbosacral spine X-ray showed no evidence of fractures but mild degenerative disc disease in L2-3 and T12-L1, with an unremarkable MRI of the lumbosacral spine as well. MRI of the pelvis demonstrated increased signals in the paraspinal muscle and bilateral abductor muscles, suggesting myositis vs. atrophy from neuritis or neuropathy (Figure 2). A colonoscopy found a 3 mm polyp in the ascending colon with hyperplastic features but negative for any malignancy or dysplasia. CSF findings showed slightly elevated glucose levels of 84 mg/dL and an elevated protein level of 148 mg/dL. All other CSF findings were unremarkable (Table 3).

**Table 2:** Autoimmune antibody findings. ANA: antinuclear antibody, ANCA: antineutrophilic cytoplasmic antibody, IFA: immunofluorescence assay, RNP: ribonucleoprotein antibody, SPEP: serum protein electrophoresis.

Antibody	Value	Reference range
ANA	Negative	Negative
ANCA	Negative	Negative
Complement C3	98	(Normal 82-185 mg/dL)
Complement C4	19	(Normal 15-53 mg/dL)
DNA (ds) antibody Crithidia IFA	Negative	Negative
Myeloperoxidase antibody	<1	<1
Proteinase 3 antibody	<1	<1
Sjogren’s Ab (SS-A)	<1	<1
Sjogren's Ab (SS-B)	<1	<1
Smith antibody	<1	<1
RNP antibody	<1	<1
SPEP protein	6.5 g/dL	6.1-8.1 g/dL
SPEP albumin	4.4 g/dL	3.8-4.8 g/dL
SPEP alpha-1 globulin	0.3 g/dL	0.2-0.3 g/dL
SPEP alpha-2 globulin	0.7 g/dL	0.5-0.9 g/dL
SPEP beta-1 globulin	0.3 g/dL	0.4-0.6 g/dL
SPEP beta-2 globulin	0.2 g/dL	0.2-0.5 g/dL
SPEP gamma globulin	0.6 g/dL	0.8-1.7 g/dL

**Table 3:** CSF findings.

CSF parameter	CSF value	CSF reference range
Volume	15 mL	
Color	Colorless	Colorless
Clarity	Clear	Clear
WBC count	0/mcL	<5/mcL
RBC count	78/mcL	
Neutrophil %	0%	
Lymphocyte %	50%	
Monocyte %	50%	
Glucose level	84 mg/dL	40–75 mg/dL
Protein level	148 mg/dL	15-45 mg/dL
Oligoclonal bands IgG	Absent	Absent

Based on the MRI of the pelvis, a nerve conduction study (NCS) and electromyography (EMG) were performed to further differentiate whether the patient had neuropathy versus myositis. NCS shows severe lower limb sensory-motor neuropathy with absent peroneal, tibial motor, and sural sensory potentials. Mild to moderate right ulnar and median motor demyelinating neuropathy was also noted in the bilateral upper extremities (Tables 4, 5). EMG depicted moderate to severe active denervation in the bilateral tibialis anterior, peroneus longus, and left vastus medialis, with no recruitment. Minimal recruitment was noted in the left rectus femoris, with moderate chronic denervation in the bilateral gastrocs and right vastus medialis (Table 6). The EMG of the bilateral upper limb muscles was unremarkable. Based on the laboratory, imagining, and clinical findings, a diagnosis of DLSRPN was made.

**Table 4:** Motor nerve conduction study.

Left median–Abductor pollicis brevis (APB)									
Nerve/site	Muscle	Latency (ms)	Amplitude (mV)	Duration (ms)	Segments	Distance (cm)	Lat Diff (ms)	Velocity (m/s)	Temp (°C)
Wrist	APB	4.95	8.7	6.61	Wrist-APB	7			29.4
Ref.		<4.40	>4.5		Ref.				
Elbow	APB	9.01	8.0	7.08	Elbow-wrist	21	4.06	52	29.4
Ref.					Ref.			>49	
Left ulnar–Abductor digiti minimi (ADM)									
Nerve/site	Muscle	Latency (ms)	Amplitude (mV)	Duration (ms)	Segments	Distance (cm)	Lat diff (ms)	Velocity (m/s)	Temp (°C)
Wrist	ADM	3.33	8.0	7.24	Wrist-ADM	7			28.9
Ref.		<4.20	>4.5		Ref.				
B. Elbow	ADM	7.55	6.3	7.81	B. Elbow–wrist	21	4.22	50	28.8
Ref.					Ref.			>49	
A. Elbow	ADM	9.32	6.3	7.45	A. Elbow–B. Elbow	10	1.77	56	28.5
Ref.					Ref.			>49	
					A. Elbow-wrist		5.99		28.5
Right median–Abductor pollicis brevis (APB)									
Nerve/site	Muscle	Latency (ms)	Amplitude (mV)	Duration (ms)	Segments	Distance (cm)	Lat diff (ms)	Velocity (m/s)	Temp (°C)
Wrist	APB	6.25	5.4	7.03	Wrist-APB	7			28.3
Ref.		<4.40	>4.5		Ref.				
Elbow	APB	10.89	4.7	7.55	Elbow-wrist	21	4.64	45	28.3
Ref.					Ref.			>49	
Right ulnar–Abductor digiti minimi (ADM)									
Nerve/site	Muscle	Latency (ms)	Amplitude (mV)	Duration (ms)	Segments	Distance (cm)	Lat diff (ms)	Velocity (m/s)	Temp (°C)
Wrist	ADM	3.65	9.6	6.35	Wrist–ADM	7			28.1
Ref.		<4.20	>4.5		Ref.				
B. Elbow	ADM	8.54	8.3	6.77	B. Elbow-wrist	21	4.90	43	27.9
Ref.					Ref.			>49	
A. Elbow	ADM	11.04	7.1	7.03	A. Elbow-B. Elbow	10	2.50	40	27.9
Ref					Ref.			>49	
					A. Elbow-wrist		7.40		27.9
Right peroneal–Extensor digitorum brevis (EDB)									
Nerve/site	Muscle	Latency (ms)	Amplitude (mV)	Duration (ms)	Segments	Distance (cm)	Lat Diff (ms)	Velocity (m/s)	Temp (°C)
Ankle	EDB	NR	NR	NR	Ankle-EDB	9			27.3
Ref.		<6.20	>2.4		Ref.				
					Pop fossa–ankle		NR		
Right tibial–Abductor hallucis (AH)									
Nerve/site	Muscle	Latency (ms)	Amplitude (mV)	Duration (ms)	Segments	Distance (cm)	Lat diff (ms)	Velocity (m/s)	Temp (°C)
Ankle	AH	NR	NR	NR	Ankle–AH	8			27.3
Ref.		<6.00	>5.0		Ref.				
Left peroneal–Extensor digitorum brevis (EDB)									
Nerve/site	Muscle	Latency (ms)	Amplitude (mV)	Duration (ms)	Segments	Distance (cm)	Lat Diff (ms)	Velocity (m/s)	Temp (°C)
Ankle	EDB	NR	NR	NR	Ankle–EDB	9			29.6
Ref.		<6.20	>2.4		Ref.				
					Pop fossa–ankle		NR		
Left tibial–Abductor hallucis (AH)									
Nerve/site	Muscle	Latency (ms)	Amplitude (mV)	Duration (ms)	Segments	Distance (cm)	Lat diff (ms)	Velocity (m/s)	Temp (°C)
Ankle	AH	NR	NR	NR	Ankle–AH	8			29.3
Ref.		<6.00	>5.0		Ref.				

**Table 5:** Sensory nerve conduction (SNC) study. NR: no response.

Right median–Digit II (antidromic)										
Nerve/sites	Rec. site		Peak lat (ms)		Amplitude (μV)	Segments		Distance (cm)		Temp (°C)
Wrist	Dig II		5.83		9.7	Wrist–Dig II		14		27.6
Ref.			<3.80		>10.0	Ref.				
Right ulnar–digit V (antidromic)										
Nerve/sites	Rec. site		Peak lat (ms)		Amplitude (μV)	Segments		Distance (cm)		Temp (°C)
Wrist	Dig V		4.74		13.7	Wrist–Dig V		14		27.5
Ref.			<3.80		>9.0	Ref.				
Right median, radial–thumb comparison										
Nerve/sites	Rec. site		Peak lat (ms)		Amplitude (μV)	Segments		Distance (cm)		Temp (°C)
Median wrist	Thumb		4.90		7.5	Median wrist–thumb		10		27.4
Ref.			<3.0		>9.0	Ref.				
Radial wrist	Thumb		3.23		4.4	Radial wrist–thumb		10		27.4
Ref.			<3.00		>4.0	Ref.				
						Median wrist–radial wrist				27.4
Left median–digit II (antidromic)										
Nerve/sites	Rec. site		Peak lat (ms)		Amplitude (μV)	Segments		Distance (cm)		Temp (°C)
Wrist	Dig II		5.47		7.5	Wrist–dig II		14		27.4
Ref.			<3.80		>10.0	Ref.				
Left ulnar–digit V (antidromic)										
Nerve/sites	Rec. site		Peak lat (ms)		Amplitude (μV)	Segments		Distance (cm)		Temp (°C)
Wrist	Dig V		4.64		3.4	Wrist–dig V		14		27.4
Ref.			<3.80		>9.0	Ref.				
Left sural–ankle (calf)										
Nerve/sites	Rec. site		Peak lat (ms)		Amplitude (μV)	Segments		Distance (cm)		Temp (°C)
Calf	Ankle		NR		NR	Calf–ankle		14		29.3
Ref.			<4.40		>4.0	Ref.				
Right sural–ankle (calf)										
Nerve/sites	Rec. site		Peak lat (ms)		Amplitude (μV)	Segments		Distance (cm)		Temp (°C)
Calf	Ankle		NR		NR	Calf–ankle		14		28
Ref.			<4.40		>4.0	Ref.				
F wave										
Nerve		F lat (ms)		Ref. (ms)			M lat (ms)		F-M lat (ms)	
Left ulnar–ADM		32.6		<32.0			3.3		29.3	
Right ulnar–ADM		38.0		<32.0			4.4		33.6	

**Table 6:** Electromyography (EMG) study. MUAP: motor unit action potential, IA: insertion activity, PSW: positive sharp waves.

			Spontaneous				MUAP			Recruitment
Muscle	Nerve	Roots	IA	Fib	PSW	Fasc	Amp	Polyphasic	Dur.	Pattern
R. Deltoid	Axillary	C5–C6	N	None	None	None	N	None	N	N
R. Biceps Brachii	Musculocutaneous	C5–C6	N	None	None	None	N	None	N	N
R. Triceps Brachii	Radial	C6–C8	N	None	None	None	N	None	N	N
R. Flexor Carpi Radialis	Median	C6–C7	N	None	None	None	N	None	N	N
R. Extensor Digitorum Communis	Radial	C7–C8	N	None	None	None	N	None	N	N
R. First Dorsal Interosseous	Ulnar	C8–T1	N	None	None	None	N	None	N	N
R. Abductor Pollicis Brevis	Median	C8–T1	N	None	None	None	N	None	N	N
L. Deltoid	Axillary	C5–C6	N	None	None	None	N	None	N	N
L. Biceps Brachii	Musculocutaneous	C5–C6	N	None	None	None	N	None	N	N
L. Triceps Brachii	Radial	C6–C8	N	None	None	None	N	None	N	N
L. Flexor Carpi Radialis	Median	C6–C7	N	None	None	None	N	None	N	N
L. Extensor Digitorum Communis	Radial	C7–C8	N	None	None	None	N	None	N	N
L. Abductor Pollicis Brevis	Median	C8–T1	N	None	None	None	N	None	N	N
L. First Dorsal Interosseous	Ulnar	C8–T1	N	None	None	None	N	None	N	N
R. Tibialis Anterior	Deep Peroneal (Fibular)	L4–L5	N	2+	4+	None				No activity
R. Gastrocnemius	Tibial	S1–S2	N	None	None	None	SI Inc	None	N	Reduced
R. Vastus Medialis	Femoral	L2–L4	N	None	None	None	SI Inc	None	N	N
R. Rectus Femoris	Femoral	L2–L4	N	None	None	None	N	None	N	N
R. Peroneus Longus	Perineal	L5–S1	N	2+	3+	None				No activity
L. Tibialis Anterior	Deep Peroneal (Fibular)	L4–L5	N	1+	3+	None				No activity
L. Gastrocnemius	Tibial	S1–S2	N	None	None	None	SI Inc	None	N	N
L. Vastus Medialis	Femoral	L2–L4	N	2+	2+	None				No activity
L. Rectus Femoris	Femoral	L2–L4	N	None	None	None	N	None	N	Reduced
L. Peroneus Longus	Perineal	L5–S1	N	2+	2+	None				No activity
L. Extensor Digitorum Brevis	Tibial	L5–S1	N	None	None	None	N	None	N	N

The patient was started on gabapentin (1200 mg three times daily) for the management of his bilateral leg pain, prednisone 40 mg daily, physical therapy, and continuation of his IVIG treatment for five days. Shortly after, he was noted to show signs of mild improvement and had been able to use a front-wheel walker, yet still had ambulation, endurance, and strength deficits. The patient continued to walk with physical therapy and was discharged home with prednisone 40 mg daily and mycophenolate mofetil 250 mg twice daily with an outpatient neurology follow-up. On follow-up, the patient noted some improvement in the proximal muscles of his right lower extremity and only pain in his left. Mycophenolate mofetil was discontinued by the patient since he was unable to tolerate the gastrointestinal upset. He was then recommended to follow-up once more to monitor the side effects of prednisone use and continue his physical therapy. At a later visit, the patient had blood work done and noted worsening HgA1c of 8.8%; however, he could extend his legs more with his right than with his left and had shown significant improvement.

## Discussion

DLSRPN is a rare type of neuropathic disease that is typically diagnosed by exclusion due to the low incidence of this disease and its non-specific clinical presentation [2]. Even though DLSRPN is generally diagnosed clinically, more common diseases should be ruled out before making such a diagnosis. A differential diagnosis should be tailored to the patient's history and clinical symptoms. Further investigation via urine toxicology, blood work, and lab imaging should help guide the diagnosis.

Due to our patient initially presenting with proximal bilateral lower extremity, weakness that progressed to his upper extremity, a concern about Guillain-Barré was noted. Unfortunately, GQ1B or GD1B were unable to be ordered for further diagnosis. However, a slight elevation of protein in the CSF slightly increased the concern for Guillain-Barré (Table 3). Although the presentation and timing were unusual, especially since the patient didn’t have any bacterial or viral illnesses and had symptoms of muscle weakness, paralysis, and pain for months prior, the concern for respiratory paralysis had to be considered. As a precaution, the patient was started on IVIG treatment, but further investigation was warranted.

Chronic inflammatory demyelinating polyneuropathy (CIDP) was likely based on the patient’s presentation of a progressive symmetrical sensorimotor disease in the proximal muscles. However, this diagnosis was noted to be unlikely based on the NCS findings. The NCS criteria for CIDP, which requires a slowing conduction velocity in at least two motor nerves to less than 70% of the lower limit of normal, have not been met. Only the right ulnar F wave is prolonged; as such, this was not enough to fulfill the diagnosis criteria [7].

Metastatic lung cancer or other malignancies were strongly considered due to unintentional weight loss and smoking history. Since the patient had complaints and concerns about weakness and pain in the bilateral lower and upper extremities, there was a possibility of tumors causing nerve compression, resulting in the patient’s symptoms. Yet, a chest x-ray, MRI of the lumbosacral spine, and colonoscopy were unremarkable for any types of metastases or malignancies.

Due to the patient’s history of having perineal abscesses and being wheelchair-bound, an abscess causing a mass effect or compression in the lumbar spine was considered. However, this was unlikely since the patient had unremarkable CBC and vitals. The physical exam also didn’t reveal any ulcers or masses that could be appreciated. Regardless, an MRI of the lumbosacral spine was ordered and showed no signs of abscess.

Vitamin B1, B12, and folate could have caused this patient's neuropathy. However, the patient denied any alcoholic history, and the urinary toxicology screen was unremarkable. The patient’s clinical presentation also showed no signs of wet beri beri or Wernicke’s encephalopathy. The diagnosis of dry beri beri by thiamine deficiency resulting in peripheral neuropathy was possible, but since the patient had no other signs of thiamine deficiency, such a diagnosis would be unlikely. This is particularly true when considering that such a diagnosis wouldn’t explain the patient’s CSF results (Table 3), MRI findings (Figure 2), or improvement on steroids. The patient’s MCV was noted to be in the 80's, further decreasing the likelihood of the diagnosis. As a consequence, alcoholic polyneuropathy can also be ruled out, based on what was stated above.

Toxin-related neuropathy secondary to mercury, lead, or other heavy metals could have contributed to this patient’s symptoms. However, the patient would have developed other symptoms alongside peripheral neuropathy. Some common symptoms vary depending on the metal. In the case of lead poisoning, the patient would also develop neuropsychiatric, hematological, gastrointestinal, etc. The patient denies having any sort of exposure to such toxins.

Isolated amyloid light (AL) chain amyloidosis-related to peripheral neuropathy was also considered since prior studies had shown the disease presenting with isolated lumbosacral radiculoplexus neuropathy. Such a disease would have also shown lower limb pain and sensory disturbances that would later develop into weakness without causing any autonomic dysfunction. The patients in those studies would also develop urinary symptoms and orthostatic hypotension. Lab findings in this disease have also shown albuminocytologic dissociation [8,9]. This diagnosis was considered, however, deemed unlikely since it wouldn’t have explained the MRI findings (Figure 2). Although only a few cases of isolated AL amyloidosis-related peripheral neuropathy have been observed, patients with such a rare disease usually require resection and immunotherapy and wouldn’t have improved with simply prednisone treatment, making the diagnosis even far less likely [9].

Even though an MRI of the pelvis shows increased signals in the paraspinal muscle, suggesting myositis, autoimmune titers have made autoimmune-related myositis, such as systemic lupus erythematosus, mixed connective tissue disease, and others (Table 2). The patient also had no elevation of creatine kinase. Urine toxicology had ruled out any drug-related causes. Serum protein electrophoresis (SPEP) showed no IgG or IgA monoclonal spikes (Table 2), making multiple myeloma unlikely. A lumbar puncture had made multiple sclerosis less probable due to a lack of oligoclonal IgG bands, and the patient denies any symptoms that would lead to such a diagnosis. With all the other differential diagnoses deemed improbable, a diagnosis of DLRPN was made.

With an estimated incidence of DLRPN of 2.79 per 100,000 people per year [10] and affecting 1% of diabetic patients [6], it is understandable why this disease is overlooked. Regardless, physicians should be aware of how to properly treat this disease once it is diagnosed. However, before making a diagnosis of DLRPN, more severe life-threatening diseases should be ruled out. History, physical exam, blood work, and imaging are paramount to excluding other diseases due to the non-specific symptoms of DLRPN. Treatment can be a difficult course of action since there have been no treatments proven to be effective measures. The symptomatic treatment of pain with gabapentin with physical therapy, exercise, and gait training has shown some clinical improvement in this case. Antidepressants with effects on neuropathic pain are also highly recommended. Even so, there is a lot of uncertainty when it comes to starting immunomodulatory therapies. Our patient was noted to have improved symptoms after starting steroids, but other studies have shown no clear evidence that immunomodulatory treatment has any success [11]. In fact, a randomized trial noted that when comparing a placebo to pulse methylprednisolone therapy, no remarkable benefit was present [12]. Conversely, some studies reported that patients showed signs of improvement with immunomodulatory treatments such as steroids or IVIG [13-15]. However, the studies were limited by the small sample size and the lack of a control group when comparing the treatments to a natural rate of recovery. Despite the ambiguity of the effects of immunomodulatory medications on DLRPN, it is crucial that these medications still be considered. When noting the fact that this disease is microscopic vasculitis [4,5], it is understandable that using glucocorticoids is crucial. Lastly, if all other medical management fails, plasma exchange can also be considered since one study showed improvement in 93% of patients [13].

## Conclusions

In general, before arriving at a diagnosis of DLRPN, a differential diagnosis of a broad range of diseases that are geared towards the patient's history and physical exam should be made. Blood work, urine toxicology, and imaging should then be done to rule out all diagnoses accordingly. Although some blood work and imaging findings can help support the diagnosis of DLRPN, such as elevated CSF protein and increased signal in the muscles in an MRI. NCS, SNC, and EMG can help further support the diagnosis because clinical exam findings are the mainstay of the diagnosis. Management should consist of alleviating the patient’s neurological symptoms, such as antidepressants or gabapentinoids, to manage the neuropathic pain. Occupation and physical therapy would be highly recommended to prevent any further pelvic muscle weakness or atrophy. Immunomodulatory medications can be considered if no contraindications exist. Glucocorticoids should be heavily considered as a first-line medication despite varying evidence, and patients should be monitored to evaluate the side effects of long-term steroid use. Lastly, plasma exchange should be considered the last resort if all other management fails. Fortunately, patients with DLRPN do tend to improve over time, but most patients don’t make a complete recovery with varying returns to function. Hopefully, by providing awareness and swift action, the progression of this disease is possible.
